# Circulating microRNAs in breast cancer: novel diagnostic and prognostic biomarkers

**DOI:** 10.1038/cddis.2017.440

**Published:** 2017-09-07

**Authors:** Rimi Hamam, Dana Hamam, Khalid A Alsaleh, Moustapha Kassem, Waleed Zaher, Musaad Alfayez, Abdullah Aldahmash, Nehad M Alajez

**Affiliations:** 1 Stem Cell Unit, Department of Anatomy, College of Medicine, King Saud University, Riyadh, Kingdom of Saudi Arabia; 2 McGill University Health Centre and RI-MUHC, Montreal, Canada; 3 Medical Oncology Unit, Department of Medicine, King Saud University, Riyadh, Kingdom of Saudi Arabia; 4 KMEB, Department of Endocrinology, University of Southern Denmark, Odense, Denmark; 5 Institute of Cellular and Molecular Medicine, Faculty of Health Sciences, University of Copenhagen, Copenhagen, Denmark; 6 Department of Anatomy, College of Medicine, King Saud University, Riyadh, Kingdom of Saudi Arabia; 7 College of Medicine Research Center, King Saud University, Riyadh, Kingdom of Saudi Arabia; 8 Prince Naif Health Research Center, King Saud University, Riyadh, Kingdom of Saudi Arabia

## Abstract

Effective management of breast cancer depends on early diagnosis and proper monitoring of patients’ response to therapy. However, these goals are difficult to achieve because of the lack of sensitive and specific biomarkers for early detection and for disease monitoring. Accumulating evidence in the past several years has highlighted the potential use of peripheral blood circulating nucleic acids such as DNA, mRNA and micro (mi)RNA in breast cancer diagnosis, prognosis and for monitoring response to anticancer therapy. Among these, circulating miRNA is increasingly recognized as a promising biomarker, given the ease with which miRNAs can be isolated and their structural stability under different conditions of sample processing and isolation. In this review, we provide current state-of-the-art of miRNA biogenesis, function and discuss the advantages, limitations, as well as pitfalls of using circulating miRNAs as diagnostic, prognostic or predictive biomarkers in breast cancer management.

## Facts

- Micro (mi)RNAs are small RNA species whose expression is often dysregulated in cancer.
- MiRNAs are present in the circulation of cancer patients and can potentially be used for disease monitoring.
- Large proportion of circulating miRNAs in cancer patients do not originate from tumors but rather reflect the body’s homeostatic response.

## Open Questions

- Are circulating miRNAs disease specific?
- What is the best approach for sample processing and detection of circulating miRNAs in breast cancer patients?
- What is the best normalization approach when quantifying circulating miRNAs?

Breast cancer is one of the most common malignant diseases in the world, with an estimated 1.5 million new cases per year.^1^ The incidence has been decreasing in the developed world;^2^ however, it remains a common cause of death in the USA and UK; Caucasian women have an estimated lifetime risk of 1 in 9.^3^

There are numerous risk factors for breast cancer, including age, family history, obesity and exposure to hormones and therapeutic radiation.^4^ Models used to estimate breast cancer risk vary depending on population characteristics; however, with the exception of hormone prophylaxis, such models are not suitable for individual patient management. The two most common types of breast cancer are ductal and lobular carcinoma. An important issue for treatment is selecting the right therapeutic modality, which is largely dependent on disease subtype. Breast cancer is currently molecularly classified based on expression of sex hormone receptors and human epidermal growth factor receptor (HER)2, which can determine diagnostic approach and treatment choice.^5^ However, other methods of classification that are based on global gene expression are gaining momentum.^6^ Molecular data – for instance, from oncotype DX breast cancer assays in lymph node-negative breast cancer – have increased our understanding of the mechanisms of chemotherapy and hormone resistance, such as the role of mutations in estrogen receptor (ER)1 in resistance to endocrine therapy.^7^

## Micro (mi)RNAs

**Figure 1:**
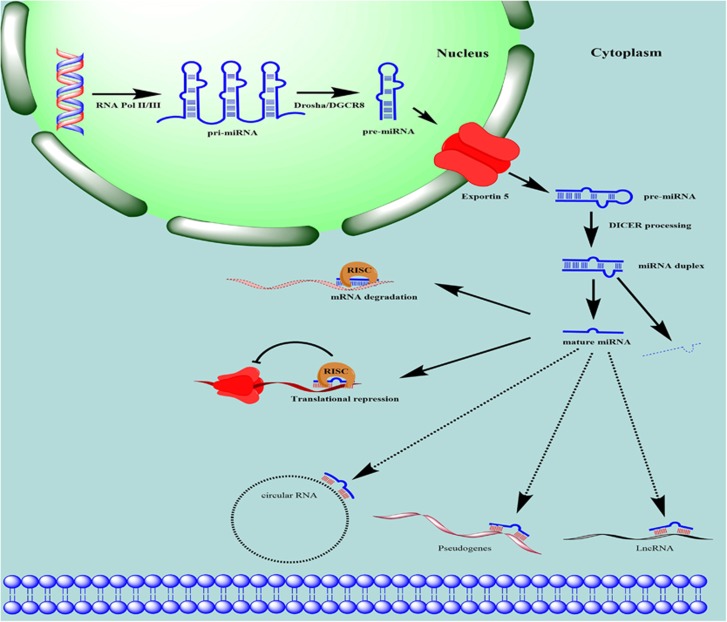
Schema depicting miRNA biogenesis and function. Primary miRNA transcript (pri-miRNA) is transcribed by RNA polymerase II/III in the nucleus, forming an elongated RNA hairpin structure that is subsequently cleaved by Drosha into a small stem-loop structure of ~70 nt, (pre-miRNA). Pre-miRNA is exported from the nucleus into the cytoplasm by exportin-5 and the loop is cleaved after the pre-miRNA is loaded onto Dicer, producing a double-stranded structure of miRNA and antisense miRNA*. The latter is typically degraded, whereas the long (~22 nt) mature miRNA strand is incorporated into the miRNA-induced silencing complex (mRISC), leading to mRNA degradation or translational repression. Mature miRNA levels are regulated via binding to ceRNAs such circular (c)RNAs, pseudogenes, and lncRNAs, which act as a sponge to prevent miRNA binding to target mRNAs

MiRNAs are short, single-stranded RNA sequences (usually 19–23 nucleotides (nts)) derived from ~70-nt precursors that control gene expression in a variety of physiological and developmental processes, thus having a critical role in post-transcriptional regulation of gene expression in a broad range of biological systems.^8, 9, 10, 11^ In humans, a single miRNA has several dozens or even hundreds of mRNA targets. Over 60% of human protein-coding genes are predicted to contain miRNA-binding sites in their 3′-untranslated region (3′-UTR).^12^ According to the miRBase database (www.mirbase.org), there are >2500 mature miRNA sequences in the human genome.^13, 14^ MiRNAs mediate the repression of target mRNAs by base paring to complementary sequences in the 3′-UTR, causing transcript destabilization, translational repression or both^15^ (Figure 1). Recent studies have reported that miRNAs also modulate gene expression by binding to other regions, including protein-coding exons,^16, 17, 18^ and can even induce gene expression in mammalian cells.^19^

## MiRNA Biogenesis and Maturation

The biogenesis of mature miRNA involves a series of biological processes (Figure 1), for review see Winter *et al.*^20^ A primary miRNA transcript (pri-miRNA) is first transcribed in the nucleus by RNA polymerase II (or sometime by RNA polymerase III), which is subsequently cleaved by Drosha into a 70-nt known as precursor miRNA (pre-miRNA).^21, 22, 23^ The pre-miRNA is exported from the nucleus to the cytoplasm by exportin-5^24, 25^ and loaded onto Dicer; the loop is then cleaved, producing a double-stranded structure composed of miRNA and antisense miRNA*.^26^ The latter is usually degraded, whereas the long (~22 nt) mature miRNA strand is incorporated into the miRNA-induced silencing complex,^27, 28^ leading to gene silencing via mRNA cleavage or translational repression depending on the degree of complementarity between the miRNA and target mRNA transcript^29, 30, 31, 32^ (Figure 1). It was recently reported that miRNAs can switch from translational repression to induction.^19^

## Regulation of miRNAs by Competing Endogenous (ce)RNAs

Although miRNAs exert their functions via direct binding to miRNA response elements (MREs) in target mRNAs, they are themselves subject to regulation when they bind to MRE-containing non-coding RNA transcripts, known as ceRNAs (Figure 1). Pseudogenes mostly originate from gene duplication and mutation and therefore lack the ability to produce functional protein.^33^ One class of pseudogenes is generated via mRNA retrotransposition (processed pseudogenes);^34^ for example, phosphatase and tensin homolog pseudogene (PTENP)1 contains many of the 3′-UTR MREs sites found in PTEN and is frequently lost in human cancer. PTENP1 was found to regulate PTEN levels by sequestering its regulatory miRNAs, including miR-19b and miR-20a.^35^

Long non-coding (lnc)RNAs are a class of RNA molecules that are longer than 200 nts. Although several lncRNAs, including X-inactive specific transcript and H19, were described decades ago, their role in gene regulation has only recently become known. For instance, the lncRNA homeobox (HOX) transcript antisense RNA (HOTAIR) was shown to interact with polycomb repressive complex 2, which is required to suppress *HOXD* gene expression.^36^ In addition, lncRNAs can function as decoys to sequester miRNAs and prevent their binding to target transcripts; for example, HOTAIR was found to regulate the expression of HER2 by acting as a miR-331-3p sponge in gastric cancer.^37^

Circular (circ)RNAs are ceRNAs generated via direct ligation of 5′ and 3′ ends of linear RNA as an intermediate during RNA splicing.^38^ CircRNAs are more stable than the linear molecule and may therefore be more efficient miRNA sponges (Figure 1). For example, ciRS-7 and sex-determining region Y act as sponges for miR-7 in neurons and miR-138 in testicular tissue.^39^

## Methods for Detecting Circulating miRNAs

Accurate quantification of circulating miRNAs in body fluids poses a number of challenges because of their low abundance and small size. However, various tools have recently been developed that overcome these obstacles, with each having advantages and limitations (Table 1). Quantitative reverse transcriptase real-time (qRT-)PCR is a widely used and highly sensitive method that requires only small amounts of input RNA.^40^ A major limitation of qRT-PCR is that it is oftentimes used to quantify the levels of a defined set of miRNAs (usually <700); as such, it cannot be used for high-throughput profiling. Microarray platforms are an alternative method for detecting circulating miRNA. The advantage of this method is the ability to simultaneously detect large numbers of circulating miRNAs;^41^ disadvantages include a low dynamic range and inability to detect novel (i.e., unannotated) miRNA species. Next-generation sequencing is another technology for detecting circulating miRNAs based on deep sequencing.^42^ This method has the advantage of being able to detect both annotated and unannotated miRNAs, although it requires large amounts of starting material and generates copious amounts of data that must be analyzed using complex bioinformatics tools. Direct quantification of circulating miRNAs in bodily fluids has become possible using the NanoString nCounter platform,^43^ which is based on a novel digital molecular barcoding technology that enables quantification of the exact copy number of miRNA species in a biological sample.^44^ However, a major limitation of this platform is that it currently can only detect up to 800 human miRNAs per slide. Given the strengths and shortcomings of each platform, selecting the appropriate one will largely depend on the available resources, type of sample, and the question being addressed.

## Sample Selection and Processing

Levels of circulating miRNAs are influenced by sample type and RNA extraction method. Serum and plasma are the most commonly used sample types for circulating miRNA detection. Hemolysis can affect the abundance of circulating miRNAs;^45, 46^ therefore, samples with obvious hemolysis should be routinely excluded from miRNA profiling studies.^41^ We have found serum to be a better sample choice for circulating miRNA studies as it is less prone to hemolysis than plasma. In addition, as miRNAs usually exist in the circulation bound to other proteins or in apoptotic bodies or exosomes, it is important to choose an RNA isolation method that will extract all miRNAs present in the desired biological fraction, such as TRIzol reagent or column-based techniques.

## Role of miRNAs in Breast Cancer

**Figure 2:**
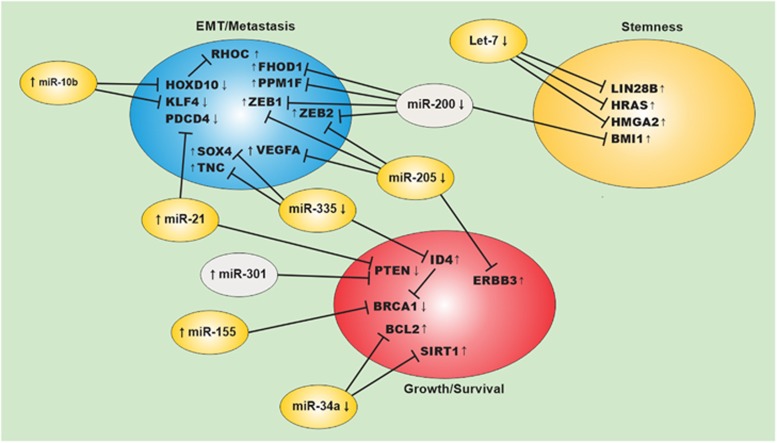
Transcriptome–miRNA interaction networks in breast cancer. Schematic representation of the interaction of commonly altered miRNAs in breast cancer and their identified mRNA targets regulating EMT/metastasis, stemness, growth and survival of breast cancer cells. ↑ indicated miRNA or gene is upregulated, whereas (↓) indicate miRNA or gene is downregulated in BC tissue. Yellow filled miRNA ovals indicate miRNAs whose expression is also altered in the circulation based on current review. RHOC, Ras homolog family member C; HOXD10, homeobox D 10; KLF4, Kruppel-like factor 4; PDCD4, programmed cell death 4; SOX4, SRY (sex-determining region Y)-box 4; TNC, Tenascin-C; FHOD1, Formin homology 2 domain containing 1; PPM1F, protein phosphatase, Mg2+/Mn2+ Dependent 1F; ZEB1, zinc-finger E-box binding homeobox 1; ZEB2, zinc-finger E-box binding homeobox 2; VEGFA, vascular endothelial growth factor A; LIN28B, Lin-28 homolog B; RAS, RAS viral oncogene homolog; HMGA2, high mobility group AT-Hook 2; BMI1, BMI1 proto-oncogene, polycomb ring finger; ID4, inhibitor of DNA binding 4, HLH protein; PTEN, phosphatase and tensin homolog; BRCA1, BRCA1, DNA repair associated; BCL2, BCL2, apoptosis regulator; SIRT1, sirtuin 1; ERBB3, Erb-B2 receptor tyrosine kinase 3

Dysregulation of miRNAs is linked to many human diseases including myocardial infarction and cardiovascular diseases,^47, 48^ diabetes, obesity^49, 50, 51^ and cancer.^52^ Various mechanisms such as DNA amplification, deletion and mutations relating to miRNA loci, epigenetic silencing or inhibition of specific miRNA processing can lead to altered miRNA expression in human cancers^53^ In this section, we describe the best-known examples of breast cancer-associated miRNAs, focusing on their involvement in various aspects of breast cancer (Figure 2).

In humans, let-7 is overexpressed in differentiated epithelial tissues and is oftentimes downregulated during tumorogenesis; it is known to target LIN28 mRNA and is itself a target of negative feedback regulation by LIN28.^44, 52^ LIN28 protein expression is upregulated in many tumors, including breast cancer.^54^ Let-7 was found to regulate breast cancer tumor-initiating cells (T-IC) through targeting HRAS and HMGA2.^55^ The miR-200 family is recognized as having a tumor-suppressor role. The family consists of five members organized as two clusters – cluster I (miR-200b/200a/429) and cluster II (miR-200c/141) on chromosomes 1 and 12, respectively,^56^ which are suppressed during epithelial-to-mesenchymal transition (EMT), an initiating step in metastasis that is associated with increased breast cancer cell motility and invasiveness.^57, 58^ MiR-200 family members were found to regulate BMI1 expression in breast cancer T-IC and suppress EMT by inhibiting zinc-finger E-box binding homeobox (ZEB)1 and ZEB2.^59, 60^ These findings were supported by another study showing that modulation of miR-200c in breast cancer cells affects cell migration and invasion.^61^ In addition, miR-200c regulates transforming growth factor *β*-induced stress fiber formation independently of the ZEB/E–cadherin axis by targeting the actin-regulatory proteins formin homology 2 domain containing (FHOD)1 and protein phosphatase, Mg^2+^/Mn^2+^-dependent (PPM)1F.^61^ MiR-10b was first discovered as an oncogenic miRNA in metastatic breast cancer cell lines;^62^ miR-10b level has been linked to malignancy in advanced-stage cancer of various types. Its expression was also upregulated in metastatic as compared with matched primary tumors.^63^ MiR-10b directly targets the *HOXD10* and Krüppel-like factor 4 genes.^62, 64^ MiR-21 is an oncogenic miRNA that inhibits several tumor-suppressor genes and thus promotes cell growth and invasion and tumor metastasis. MiR-21 is one of the most highly expressed miRNAs in breast cancer, and its upregulation is associated with tumor progression and poor prognosis.^65, 66^ MiR-21 has several targets including tropomyosin 1*α* and programmed cell death (PDCD)4.^66, 67^ MiR-21 also targets PTEN^68^ to promote MCF-7 breast cancer cell growth,^69^ as well as the tumor suppressors acidic nuclear phosphoprotein 32 family member A and SWI/SNF-related matrix-associated actin-dependent regulator of chromatin subfamily A member 4.^70^ MiR-335, which is oftentimes silences in breast cancer, inhibits metastasis by targeting the transcription factor Sry-box 4 and extracellular matrix protein tenascin-C.^71, 72^ The tumor-suppressor function of miR-335 involves reducing cell viability and promoting apoptosis by simultaneously regulating the *BRCA1* activators insulin-like growth factor 1, ER-*α*, and specificity protein 1 and the repressor inhibitor of differentiation 4.^73^ MiR-301 acts as an oncomiR in breast cancer via regulation of forkhead box F2, B-cell lymphoma 2-binding component 3, PTEN and collagen 2A1.^74^

MiR-155 is another oncogenic miRNA that regulates multiple signaling pathways related to cell growth and survival;^75^ it is known to target *BRCA1*, a human breast cancer susceptibility gene^76, 77^ that is involved in DNA repair and cell cycle progression. Other genes associated with breast cancer progression such as *suppressor of cytokine signaling 1* and *forkhead box O3a* are negatively regulated by miR-155.^78^ MiR-34a is oftentimes downregulated in breast cancer, which promotes breast cancer growth and survival through upregulation of SIRT1 and BCL2 proteins.^79, 80^ miR-205 is frequently downregulated in metastatic breast cancer. Loss of miR-205 promoted breast cancer cell growth and invasion through upregulation of Erb-B2 receptor tyrosine kinase 3, vascular endothelial growth factor A, ZEB1 and ZEB2 proteins.^81, 82, 83^

## Circulating miRNAs as Disease Biomarkers

**Figure 3:**
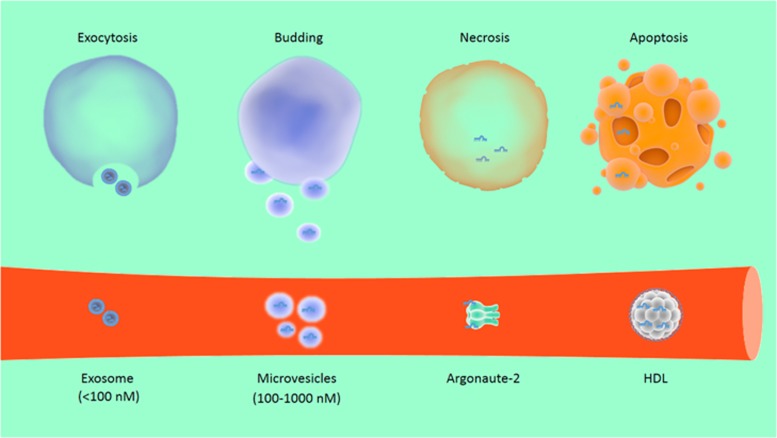
Sources and forms of circulating miRNAs. MiRNAs can be released via an active process in the form of exosomes (<100 nM) through the course of exocytosis (a process that involves fusion of the multivesicular body (MVBs) with the plasma membrane) or as microvesicles (100–1000 nM, through outward budding from the plasma membrane). Alternatively, miRNAs can be released as a result of necrosis or apoptosis (programmed cell death). Cell-free circulating miRNAs usually exist bound to ribonucleoprotein complexes (such as Argonaute-2), or high-density lipoprotein (HDL). Circulating miRNAs are also found within lipid microvesicles and exosomes

Cell-free circulating miRNAs usually exist bound to ribonucleoprotein complexes or high-density lipoprotein or they are released from cells in lipid vesicles, microvesicles, exosomes or apoptotic bodies (Figure 3).^84, 85, 86, 87^ Lipid vesicles and exosomes have critical roles in cell–cell communication.^87, 88^ Thus, circulating miRNAs may reflect homeostatic response of the organism, as well as signs of disease progression. Circulating miRNAs have been detected in the peripheral blood circulation and other body fluids.^89, 90, 91^ Owing to their stability and resistance to endogenous RNase activity, these miRNAs have been proposed as diagnostic and prognostic biomarkers for diseases, such as cancer, diabetes mellitus and neurological disorders.^92, 93, 94, 95^ Table 2 summarizes the frequently upregulated circulating miRNAs in human cancers. Elevated levels of miR-21 and -210 in the serum have been reported in patients with diffuse large B-cell lymphoma; the former is associated with relapse-free survival.^96^ Increased serum levels of various miRNAs have been linked to different human cancers – for instance, miR-141 in prostate cancer;^91^ miR-25 and miR-223 in lung cancer;^89^ miR-21, miR-92, miR-93, miR-126 and miR-29a in ovarian cancer;^97^ miR-92 and miR-17-3p in colorectal cancer;^98^ miR-92a in acute leukemia;^99^ miR-210, miR-21, miR-155 and miR-196a in pancreatic cancer;^100, 101^ miR-184 in squamous cell carcinoma of the tongue;^102^ and miR-500 in hepatocellular carcinoma.^103^

A large number of studies have reported the usefulness of miRNAs as diagnostic, prognostic, or predictive biomarkers for breast cancer. Table 3 list characteristics of the studies including number of patients included, verification of miRNAs identified using alternative methods and confirmation of the findings in an independent cohort of patients. As seen in Table 3, studies varied in their quality based on these quality criteria. In the following sections, we will discuss how circulating miRNAs have been used in the context of breast cancer biology as diagnostic, prognostic and predictive biomarkers.

### Circulating miRNAs as diagnostic biomarkers

Heneghan and colleagues^104^ assessed the diagnosis potential of a panel of seven cancer-associated miRNAs in the circulation of patients with various cancer types. The authors found that let-7a and miR-10b and -155 levels were upregulated in the majority of cancer patients, whereas circulating miR-195 level distinguished those with breast cancer from other cancer types and from normal control with a sensitivity of 88% and a specificity of 91%. The sensitivity was further increased to 94% when using a combination of circulating levels of miR-195, let-7a and miR-155. Another study explored the diagnostic potential of a panel of circulating miRNAs targeting PTEN tumor suppressor using qRT-PCR in a cohort of breast cancer patients. The preoperative levels of circulating miR-20a and -21 were higher in patients with breast cancer and benign disease compared with healthy controls, whereas levels of circulating miR-214 were able to discriminate between malignant and benign tumors and healthy subjects.^105^ Cuk *et al.*^106^ explored the diagnostic potential of a seven circulating miRNA panel (miR-127-3p, miR-148b, miR-376a, miR-376c, miR-409-3p, miR-652 and miR-801) in two cohorts of breast cancer patients. The authors observed elevated levels of these miRNAs in the circulation from breast cancer patients. MiR-127-3p, miR-148b, miR-409-3p, miR-652 and miR-801 were detected in breast cancer stages I and II, suggesting that they can be used for early diagnosis. In another study, miRNA expression profiling using plasma samples from breast cancer patients and healthy controls revealed 43 miRNAs that were differentially expressed between the two groups, with patients exhibiting higher miR-148b, miR-133a and miR-409-3p levels. miR-148b and miR-133a were also detected in breast cancer cell lines, suggesting their tumor origin.^107^ A recent study performed global profiling of circulation miRNA in patients with ER-positive early-stage breast cancer and age-matched healthy controls. The authors identified a panel of nine miRNAs (miR-15a, miR-18a, miR-107, miR-133a, miR-139-5p, miR-143, miR-145, miR-365 and miR-425) that can discriminate between patients with breast cancer and healthy controls. This panel was subsequently validated in a second cohort of patients with early-stage breast cancer.^108^ A study using the Taqman low-density array platform comparing patients with early breast cancer and healthy controls found that circulating miR-484 level was higher in patients with breast cancer, which was validated in a second cohort of patients with early-stage breast cancer.^109^ Although several of the aforementioned studies included a modest number of patients, Shimomura *et al.*^110^ conducted microarray expression profiling using sera from a cohort of 1280 patients with breast cancer, 2836 controls, 451 from patients with other cancer types and 63 from patients with non-breast benign diseases. The authors divided the samples into training and validation cohorts and identified a panel of five miRNAs (miR-1246, miR-1307-3p, miR-4634, miR-6861-5p and miR-6875-5p) that discriminated between patients with breast cancer and those with other cancer types and controls. In another study, serum miR-155, miR-19a, miR-181b and miR-24 levels were elevated in patients with early-stage breast cancer relative to healthy subjects at the time of diagnosis, and were higher in high-risk as compared with low-risk patients. Interestingly, miR-155, miR-181b and miR-24 expression declined after surgical resection whereas that of miR-19a decreased post-therapy.^111^

However, one limitation of the above-mentioned studies is that the origin of detected circulating miRNAs has not been verified and in particular, the contribution of breast cancer tissue to the identified circulating miRNAs is not known. A number of studies examined the association between the expression of selected miRNA panels in breast cancer tissues and their correlation with circulating miRNAs. For instance, global profiling of miRNA expression in breast cancer tumor tissue, normal tissue and serum samples obtained from patients and from healthy controls revealed significant deregulation in the expression level of several miRNAs in both tissue and circulation. In particular, miR-1, miR-92a, miR-133a and miR-133b were the most prominently upregulated diagnostic markers in breast cancer sera, which was subsequently validated in an independent cohort of patients with breast cancer.^112^ Matamala *et al.*^113^ performed global miRNA expression profiling using paraffin-embedded tissue from patients with breast cancer and samples from healthy controls; in two independent cohorts of breast cancer *versus* control, they identified and validated four miRNAs (miR-505-5p, miR-125b-5p, miR-21-5p and miR-96-5p) that were significantly overexpressed in tissue and circulation of pretreated patients with breast cancer. In another study, Li *et al.*^114^ assessed the expression of Let-7c in breast cancer tissue compared with adjacent para-carcinoma control tissue. The authors reported significant downregulation in Let-7c in breast cancer. The tissue findings were subsequently verified using sera of patients with breast cancer that revealed lower levels of let-7c compared with serum levels in healthy controls. Wang *et al.*^115^ assessed the diagnostic potential of circulating miR-182 in breast cancer patients. High serum levels of circulating miR-182 measured by qRT-PCR were detected in breast cancer patients as compared with healthy controls and miR-182 was also overexpressed in breast cancer tissue, suggesting that it is potential use as a diagnostic biomarker. Although in the above-mentioned studies a good concordance was observed in miRNAs profile detected in breast cancer tumor tissue and in the circulation, some studies suggested a non-tumor origin of circulating miRNAs. Waters and colleagues^116^ used a murine model of breast cancer to assess changes in circulating miRNA expression during tumor progression. Of particular interest, circulating miR-138 was upregulated in the murine xenograft model of breast cancer, whose upregulated expression was subsequently validated in the sera from patients with breast cancer. Interestingly, the authors did not observe any change in miR-138 levels in breast cancer tissues itself.

miRNA expression profiling of breast cancer tissue revealed that several miRNAs exhibited expression pattern associated with breast cancer molecular subtype, ER status or other pathological features.^117, 118^ Therefore, a number of studies assessed potential utilization of circulating miRNAs in breast cancer disease stratification. In one study, Zhu *et al.*^119^ assessed the expression of circulating miR-16, miR-145 and miR-155 in a cohort of breast cancer patients compared with those in healthy controls. Although there was no difference in the expression of this miRNA panel in breast cancer, miR-155 was found to be highly expressed in progesterone (PR)-positive patients. Wang and colleagues^120^ correlated the expression of selected panel of miRNAs in breast cancer tissues and matched serum samples. The authors observed miR-21, miR-106a and miR-155 levels to be elevated, whereas those of miR-126, miR-199a and miR-335 to be reduced in tumor specimens relative to normal tissue. Interestingly, circulating levels of miR-21, miR-126, miR-155, miR-199a and miR-335 were associated with histological tumor grade and sex hormone receptor expression. Recently our group isolated and performed an enrichment step before global expression profiling of circuiting miRNAs in breast cancer patients compared with normal controls. We identified a novel panel of nine circulating miRNAs (miR-4270, miR-1225-5p, miR-188-5p, miR-1202, miR-4281, miR-1207-5p, miR-642b-3p, miR-1290 and miR-3141) that was upregulated in patients with breast cancer, and whose expression was correlated with cancer stage and molecular subtype.^41^

### Circulating miRNAs as prognostic biomarkers

Circulating miRNAs can also serve as prognostic biomarkers in breast cancer patients (Table 3). A prognostic biomarker should indicate patient’s outcome, for example, disease recurrence or disease progression, independent of treatment received. In one study, serum levels of melanoma-associated antigen-A1, -A2, -A3 and -A12 and CCCTC-binding factor-like mRNA, as well as that of let-7b were higher in patients with invasive breast cancer compared with those with non-invasive tumors, benign breast disease or healthy controls. In this study, miR-202 overexpression was positively correlated with reduced overall survival.^121^ In another study, Mangolini and colleagues^122^ used droplet digital PCR to assess the prognostic value of a five circulating miRNA (miR-10b-5p, miR-145-5p, miR-148b-3p, miR-425-5p and miR-652-3p) panel chosen based on prior circulating miRNA expression profiling study. The authors reported that serum levels of miR-148b-3p and miR-652-3p were lower, whereas higher expression of miR-10b-5p correlated with poor prognosis in two cohorts of breast cancer patients. A recent study investigated the prognostic value of circulating miRNAs in patients with primary triple-negative breast cancer (TNBC). The authors conducted genome-wide miRNA expression profiling using serum from TNBC patients, which revealed a four-miRNA signature (miR-18b, miR-103, miR-107 and miR-652) that could predict tumor relapse and overall survival.^123^

Several studies has also investigated the potential correlation between miRNA profile expression in the circulation and in breast cancer metastastic tissue and their possible use to diagnose generalized metastatic disease. Based on the observation that miR-10b and miR-373 are overexpressed in breast cancer lymph node metastases, circulating levels of miR-10b and miR-373 were assessed in the sera for their potential utilization as biomarker for detecting breast cancer lymph node metastases. The authors reported higher levels of miR-10b and miR-373 in plasma from preoperative breast cancer patients with lymph node metastasis compared with patients without metastasis and normal controls.^124^ In another study, serum levels of four breast cancer-associated miRNAs (miR-10b, miR-34a, mi-R141 and miR-155) were measured in patients with primary or metastatic breast cancer, and healthy controls using qRT-PCR. Increased expression of circulating miR-34a was correlated with tumor stage, whereas upregulation of miR-10b, miR-34a and miR-155 was associated with the presence of metastases.^125^ Shaker *et al.*^126^ assessed the expression of four miRNAs in the sera of female patients with breast cancer and healthy controls and reported increased levels of miR-29b-2, miR-155, miR-197 and miR-205 in patients with breast cancer and the levels of these miRNAs correlated with tumor grade (T3 *versus* T2) and the presence of lymph node metastases (N3 *versus* N2), whereas expression of only miR-155 and miR-205 correlated with the presence of distant metastases. miRNA expression profiling performed on breast cancer tissue, serum samples from patients with early-stage breast cancer and healthy controls revealed that the level of miR-92a was reduced whereas that of miR-21 was increased in the tissue and serum of patients with early-stage breast cancer relative to controls. Furthermore, serum miR-92a and -21 levels were correlated with tumor size and the presence of lymph node metastases.^127^ In addition, miRNA profiling of recurrent, non-recurrent and healthy controls identified 22 miRNAs, which were subsequently validated in an independent cohort of non-recurrent and recurrent breast cancer patients’ sera. Upregulation of miR-21-5p, miR-375, miR-205-5p and miR-194-5p and downregulation of miR-382-5p, miR-376c-3p and miR-411-5p have been linked to breast cancer recurrence.^128^ Another study has reported an association between the expression of cell-free exosomal miRNAs circulating in serum and the molecular subtypes of breast cancer. The authors measured the expression levels of six circulating miRNAs (miR-10b, miR-17, miR-34a, miR-93, miR-155 and miR-373) in the sera of patients with primary (M0), and metastatic (M1) breast cancer and those of healthy controls. The authors observed significant differences in the expression levels of circulating miR-34a, miR-93 and miR-373 between the patients with M0 breast cancer and healthy controls, whereas the levels of miR-17 and miR-155 were significantly higher in the M0 compared with those in the M1 group. Elevated levels of miR-373 were associated with HER2-negative status of the primary tumor, whereas levels of miR-17 and miR-34a correlated with PR or ER status.^129^

### Circulating miRNAs as predictive biomarkers

Only few studies have investigated the value of circulating miRNAs in breast cancer patients as predictive biomarkers for treatment response. In one study, expression of four breast cancer-associated miRNAs (miR-10b, miR-34a, miR-125b and miR-155) was profiled in the sera from breast cancer patients with invasive ductal carcinoma and preoperative neoadjuvant chemotherapy before treatment and normal controls. Among the studied miRNAs, only miR-125b exhibited higher expression level in non-responder breast cancer patients suggesting possible correlation between miR-125b expression in the circulation and breast cancer chemotherapeutic resistance.^130^ In an independent study, Wu and colleagues^42^ performed deep sequencing of circulating miRNAs on pre-treatment sera obtained from a cohort of stages II–III locally advanced breast cancer patients who received neoadjuvant chemotherapy followed by surgical resection of the tumor. The authors observed that reduced level of miR-375 and elevated levels of miR-122 were able to discriminate between relapsed and non-relapsed patients, whereas elevated levels of miR-375, miR-184, miR-1299 and miR-196a and reduced levels of miR-381, miR-410 and miR-1246 were observed in good responder to neoadjuvant chemotherapy. The authors subsequently validated miR-122 in a second cohort of stages II–III BC patients and demonstrated significant association between elevated expression of circulating miR-122 and patient relapse, suggesting potential utilization of miR-122 and miR-375 in predicting response to chemotherapy in BC patients and relapse. Sun and colleagues^131^ used qRT-PCR to assess the expression of miR-155 in the sera from breast cancer patients compared with healthy individuals and reported elevated circulating levels of miR-155 in breast cancer. Interestingly, the levels of miR-155 in the serum decreased after surgery and four cycles of chemotherapy suggesting potential utilization of miR-155 as an indicator for treatment response.

## Limitations of Using miRNAs as Breast Cancer Biomarkers

The establishment of an accurate and reliable panel of circulating miRNAs for breast cancer diagnosis, prognosis and prediction of treatment response, is challenging at nearly every step from sample collection and processing to data analysis.^132^ A major limitation to using circulating miRNAs as biomarkers is their low abundance, which hampers their detection using standard miRNA profiling techniques such as microarrays. Modified approaches have been proposed as a solution: for example, we recently developed a new strategy that relies on miRNA isolation and enrichment before global expression profiling.^41^ Another important issue is sample selection and processing. The majority of studies use serum, or plasma. We found that serum is a better choice to avoid drawbacks of excluding a large number of samples because of the presence of hemolysis. Notably, circulating miRNA levels are higher in serum than in plasma, implying potential interference by platelet and white blood cell during sample preparation.^133^ It is therefore important to use the same type of material (for patients and controls), avoid samples showing signs of hemolysis and use a standardized protocol of sample collect and processing. Patient selection and classification is a critical issue for clinical studies. A number of studies have reported fluctuations in circulating miRNA levels in response to chemotherapy.^134, 135, 136^ To eliminate this problem, patients’ treatment regimen must be considered, or else blood samples must be collected before chemotherapy. Another important factor is the choice of platform for measurement of miRNA level. It is clear that the majority of studies listed in Table 3 are qRT-PCR-based. Although this method is more sensitive and less costly than others, a major limitation is the inability to detect novel miRNAs; indeed, most studies used a pre-existing circulating miRNA panel or screened for miRNAs that have been detected in tissue. Finally, there is a lack of reliable housekeeping circulating miRNAs for normalization of expression levels, which can change with physiological and pathological status. As such, other approaches for normalization have been used, such as using equal amounts of starting material (serum or plasma)^41^ or a synthetic spike-in control, which was found to be more reliable than endogenous miRNAs for data normalization.^137^

As observed in the above appraisal of the published studies, there is a minimal overlap in the identified miRNA panels among different studies, which reflects a complex biology of miRNA expression in BC patient’s circulation. Breast cancer is a heterogeneous group of diseases with diverse biological behaviors that correspond to heterogeneous cancer tissue structure and gene expression profile. The discrepancy in the identified circulating miRNA signatures reported by different groups, may be caused by the heterogeneity of the disease and its clinical presentation. The variability may also be caused by the significant contribution of many tissues to the circulating miRNAs. Circulating exosomes and microvesicles are carrier of miRNAs in the circulation and their content and biological functions are dependent on their cell of origin;^138^ a potential solution to obtain a breast cancer-specific profile is to enrich tumor-specific miRNAs through isolation of circulating microvesicles using markers specific to the tissue of origin. For instance, epithelial cell adhesion molecule (EpCAM)-positive microvesicles were isolated from ovarian cancer for miRNA profiling.^139^

## Conclusions

Circulating miRNAs hold a great promise as diagnostic, prognostic or predictive biomarkers in the clinical management of patients with breast cancer. In our review of the current state of knowledge in the field, we observed little consistency with respect to the circulating miRNA panels identified by different research groups, hence currently we do not have clinically useful panel of circulating miRNA to be used in the oncology practice. This is in part because of variations in patient selection and techniques used to isolate and measure circulating miRNAs, their low abundance, the effects of therapy, and concurrent diseases, inadequate sample sizes, inadequate statistical analysis, and insufficient numbers of validation studies testing their clinical utilization. A number of issues related to sample collection, method of measurements and normalization are still in need for standardization and streamlining, whereas a number of approaches are currently under development to enhance detection sensitivity and specificity and improve the clinical applications of miRNAs. Multi-center global profiling studies may provide useful data for identifying diagnostic, prognostic, or predictive circulating miRNA panels. Although these issues may hamper the clinical use of miRNA profiling in clinical practice, their use as research tools to understand and possibly target cancer cells and cancer stem cells are currently rich areas for clinical investigation.

## Figures and Tables

**Table 1:** Commonly used methods for quantifying circulating miRNAs

**Method**	**Advantages**	**Disadvantages**
Quantitative real-time PCR	Highly sensitive Requires small amounts of input RNA	Mostly used to quantify the level of a defined set of miRNAs
Microarray	Can simultaneously measure large numbers of circulating miRNAs	Low dynamic range Unable to detect novel unannotated miRNAs
Next-generation sequencing	Can detect both annotated and unannotated miRNAs	Requires large amounts of starting material Generates copious amounts of data requiring complex bioinformatics data analysis
NanoString nCounter	Can quantify the exact copy number of miRNA species in biological samples	Currently limited to detecting up to 800 miRNAs per sample

**Table 2:** List of selected circulating miRNAs in various human cancers

**MiRNA**	**Cancer type**	**Regulation**	**Reference**
Mir-21 and -210	B-cell lymphoma	Up	^96^
MiR-141	Prostate cancer	Up	^91^
MiR-25 and -223	Lung cancer	Up	^89^
MiR-21, -92, -93, -126, and -29a	Ovarian cancer	Up	^97^
MiR-17-3p and -92	Colorectal cancer	Up	^98^
MiR-92a	Acute Leukemia	Up	^99^
MiR-210, -21, -155, and -196a	Pancreatic cancer	Up	^100, 101^
MiR-184	Squamous cell carcinoma	Up	^102^
MiR-500	Hepatocellular carcinoma	Up	^103^

**Table 3:** Circulating miRNAs as Diagnostic, Prognostic, or predictive biomarkers in breast cancer

**Source**	**No.**	**MiRNA**	**Expression level**	**Diagnostic**	**Prognostic**	**Predictive**	**Validated**	**Platform**	**Reference**
Blood	83	MiR-195, let-7 and -155	Higher in BC patients	Yes	No	No	No	qRT-PCR	^104^
Serum	168	MiR-214	Discriminates malignant from benign tumors and healthy subjects	Yes	No	No	No	qRT-PCR	^105^
Plasma	247	MiR-127-3p, -376a, -148b, -409-3p, -652 and -801	Higher in BC patients	Yes	No	No	Yes	qRT-PCR	^106^
Plasma	137	MiR-148b, -133a, and -409-3p	Higher in BC patients	Yes	No	No	Yes	qRT-PCR	^107^
Serum	108	MiR-15a MiR-18a, -107, -425, -133a, -139-5p, -143, -145, and -365	Higher in BC patients Lower in BC patients	Yes	No	No	Yes	qRT-PCR	^108^
Serum	137	MiR-484	Higher in BC patients	Yes	No	No	Yes	qRT-PCR	^109^
Serum	1280	MiR-1246, -1307-3p, and -6861-5p MiR-4634 and -6875-5p	Higher in BC patients Lower in BC patients	Yes	No	No	Yes	Microarray; qRT-PCR	^110^
Serum	63	MiR-155, -19a, -181b, and -24	Higher in BC patients	Yes	No	No	No	qRT-PCR	^111^
Serum	164	MiR-1, -92a, -133a, and -133b	Higher in BC patients	Yes	No	No	Yes	Microarray; qRT-PCR	^112^
Plasma	197	MiR-505-5p, -125b-5p, -21-5p, and -96-5p	Higher in BC patients	Yes	No	No	Yes	qRT-PCR	^113^
Serum	90	let-7c	Lower in BC patients	Yes	No	No	No	qRT-PCR	^114^
Serum	46	MiR-182	Higher in BC patients	Yes	No	No	No	qRT-PCR	^115^
Blood	83	MiR-138	Higher in BC patients	Yes	No	No	No	Microarray; qRT-PCR	^116^
Serum	13	MiR-155	Correlates with PR status	Yes	No	No	No	qRT-PCR	^119^
Serum	68	MiR-21, -126, -155, -199a, and -335	Associated with histological tumor grade and sex hormone receptor expression	Yes	No	No	No	qRT-PCR	^120^
Serum; Plasma	46	MiR-4270, -1225-5p, -188-5p, -1202, -4281, -1207-5p, -642b-3p, -1290, and -3141	Higher in BC patients and correlates with stage and molecular subtype	Yes	No	No	Yes	Microarray; qRT-PCR	^41^
Serum	102	MiR-202 and let-7b	Higher expression in BC patients and correlates with tumor aggressive and overall survival	Yes	Yes	No	No	qRT-PCR	^121^
Serum	87	MiR-148b-3p and -652-3p MiR-10b-5p	Lower in the BC patients Higher levels correlate with poor prognosis	Yes	Yes	No	Yes	ddPCR	^122^
Serum	130	MiR-18b, -103, -107, and -652	Associated with tumor relapse and overall survival in TNBC patients	Yes	Yes	No	Yes	qRT-PCR	^123^
Plasma	60	MiR-10b and -373	Higher in breast cancer patients with LN metastasis	Yes	Yes	No	Yes	qRT-PCR	^124^
Serum	89	MiR-10b, 34a, and -155	Correlates with tumor stage and/or metastasis	Yes	Yes	No	No	qRT-PCR	^125^
Serum	100	miR-29b-2, miR-155, miR -197 and miR -205	Correlates with tumor grade and metastasis	Yes	Yes	No	No	qRT-PCR	^126^
Serum	100	MiR-92a MiR-21	Lower in BC patients, LN metastasis Higher in BC patients, LN metastasis	Yes	Yes	No	No	qRT-PCR	^127^
Serum	90	MiR-21-5p, -375, -205-5p, and -194-5p MiR-382-5p, -376c-3p, and -411-5p	Higher in recurrent BC patients Lower in recurrent BC patients	Yes	Yes	No	Yes	qRT-PCR	^128^
Serum	152	MiR-34a, -93, -373, -17, and -155	Expression correlated with metastasis and HER2, PR, and ER status	Yes	No	No	No	qRT-PCR	^129^
Serum	56	miR-125b	Higher expression in non-responsive patients	Yes	No	Yes	No	qRT-PCR	^130^
Serum	68	MiR-122 MiR-375	Lower in NR and pCR Higher in NR and pCR	No	No	Yes	Yes	DS; qRT-PCR	^42^
Serum	103	MiR-155	Higher in BC patients; decreased level after chemotherapy	Yes	No	Yes	No	qRT-PCR	^131^

Abbreviations: BC, breast cancer; ddPCR, droplet digital PCR; DS, deep sequencing; ER, estrogen receptor; HER2, human epidermal growth factor receptor 2; LN, lymph node; miRNA (miR), microRNA; PR, progesterone receptor; qRT-PCR, quantitative reverse transcriptase real-time PCR; TNBC, triple-negative breast cancer. NR, non-relapse; pCR, Pathologic complete response.
